# Interacting circadian and homeostatic processes with opportunity cost: A mathematical model of sleep with application to two mammalian species

**DOI:** 10.1371/journal.pone.0208043

**Published:** 2018-12-12

**Authors:** James H. Cardon, Eric R. Eide, Kerk L. Phillips, Mark H. Showalter

**Affiliations:** 1 Brigham Young University, Department of Economics, Provo, UT, United States of America; 2 US Congressional Budget Office, Washington, DC, United States of America; University of Southampton, UNITED KINGDOM

## Abstract

This paper introduces a new model of sleep for mammals. It extends the classic ‘two-process’ model of sleep to account for differences in external circumstances. We apply this model to previously-collected data on elephants and sloths, comparing sleep patterns in the wild with sleep patterns in captivity. We find that the model does very well in explaining sleeping patterns for both types of animals, in both the captive state and in the wild state.

## Introduction

All known forms of animal life must sleep ([1]). As with other essential biological functions like eating and breathing, insufficient sleep can lead to serious biological consequences, including death [2].

The most widely-used mathematical model of sleep, typically referred to as the two-process model, combines two types of biological processes, the circadian cycle and sleep pressure (also referred to as homeostasis) ([3], [4] and [5]). The circadian cycle is a roughly 24-hour pattern of alertness and fatigue that also leads to daily cyclical patterns of body temperature, hunger, metabolic rate, etc.

Sleep pressure increases the longer a subject is awake, with a corresponding increase in the physical desire to sleep. This combination of the circadian cycle and sleep pressure creates a deterministic prediction for sleep patterns and is widely used to explore sleep patterns in humans and animals.

The two-process model summarizes the underlying biological forces determining the sleep cycle. However, the model does not account for substantial day-to-day variation in sleep patterns. From an economic perspective, the model is incomplete in that it ignores the fact that sleep is costly because it implies forgoing other activities that contribute to well-being. Economists would say that sleep has an opportunity cost that varies substantially across individuals and over time [6].

As an example of such an opportunity cost, consider that animals in captivity tend to sleep more than their counterparts in the wild. Although biologically identical, all else equal, the cost of sleeping is much lower in captivity than in the wild because such animals have no need to forage for food or to avoid predators. That animals vary their sleep in order to take advantage of time-varying opportunities is evidence that the deterministic two-process model is incomplete.

The main contribution of this paper is to incorporate the economic concept of opportunity cost into the widely-used and deterministic two-process model of sleep. Specifically, we augment the two-process model to allow subjects to trade off gains from sleep derived from the sleep cycle with gains from alternative, productive uses of time.

We illustrate our model with an application to elephants and sloths in the wild and in captivity. For these animals, the opportunity cost of sleep can be represented by activities such as foraging for food and avoiding predators. In the context of a mathematical model of sleep, we assume animals choose to sleep or wake in order to optimize a more general objective function incorporating gains from waking activities as well as from sleep. We show that this can lead to different and better outcomes for the animals than choices conforming to mechanical models of sleep. Since the value of waking activity changes over time and across individuals, the model generates predictions about how certain variables affect sleep. We also demonstrate that the two-process model is a special case of our economic model of sleep.

The dynamics of our model are driven by key parameter values, some of which can be estimated only by fitting to data. Data on sleep patterns of animal species facing differing opportunity cost environments are scarce. We show that by incorporating differing opportunity costs of sleep, our model is able to match the sleep patterns for wild and captive elephants and sloths.

The model can be used to approximate the relative value of sleep time for these two populations for both species. This value would be nearly impossible to measure directly as it includes so many different components. Our model and identification scheme show that the opportunity cost of sleep for wild elephants is six to seven times greater than for captive ones. We find values in that same range for sloths.

Mechanistic models of sleep, like the two-process model, miss some key forces driving behavior. Animals evolved in and confront environments where the cost of sleep changes. Sleep behavior has evolved to deal with these dynamic environments. A better understanding of sleep behavior requires learning how sleep changes as its costs change. Incorporating opportunity cost into models of sleep behavior can improve understanding of sleep in both animals and humans.

## Models of sleep

### Two-process model

We now provide the details of the two-process model. In the next section we present a more general sleep model which allows for choice in the timing of sleep as a function of the opportunity cost of sleep. We take our discussion and notation from [7], but adopt a discrete-time framework rather than their continuous-time setup.

There is a baseline circadian cycle, which we will denote *y*_*t*_. This is a weighted sum of sine waves of various frequencies and will fluctuate between -1 and +1. There are upper and lower bounds related to this cycle. We denote these $H_{t}^{u}$ and $H_{t}^{l}$:

$$\begin{array}{c} \begin{array}{cc} H_{t+1}^{u} & ={\bar{H}}^{u}+ay_{t}\\ H_{t+1}^{l} & ={\bar{H}}^{l}-ay_{t}\\ & {\bar{H}}^{u}>{\bar{H}}^{l} \end{array} \end{array}$$ (1)

The homeostatic process which determines sleep versus waking is denoted *H*_*t*_. If waking it rises toward an upper asymptote of *μ* and if sleeping it falls toward a lower asymptote of zero, so that

$$\begin{array}{c} H_{t+1}=\{\begin{array}{cc} H_{t}e^{-1/\nu_{S}} & \text{if}\;\text{sleeping}\;\\ \mu +\left(H_{t}-\mu \right)e^{-1/\nu_{W}} & \text{if}\;\text{waking}\; \end{array} \end{array}$$ (2)

where *ν*_*W*_ is the constant half-life decay for the homeostatic process when awake and *ν*_*S*_ is the half-life when sleeping.

**Fig 1:**
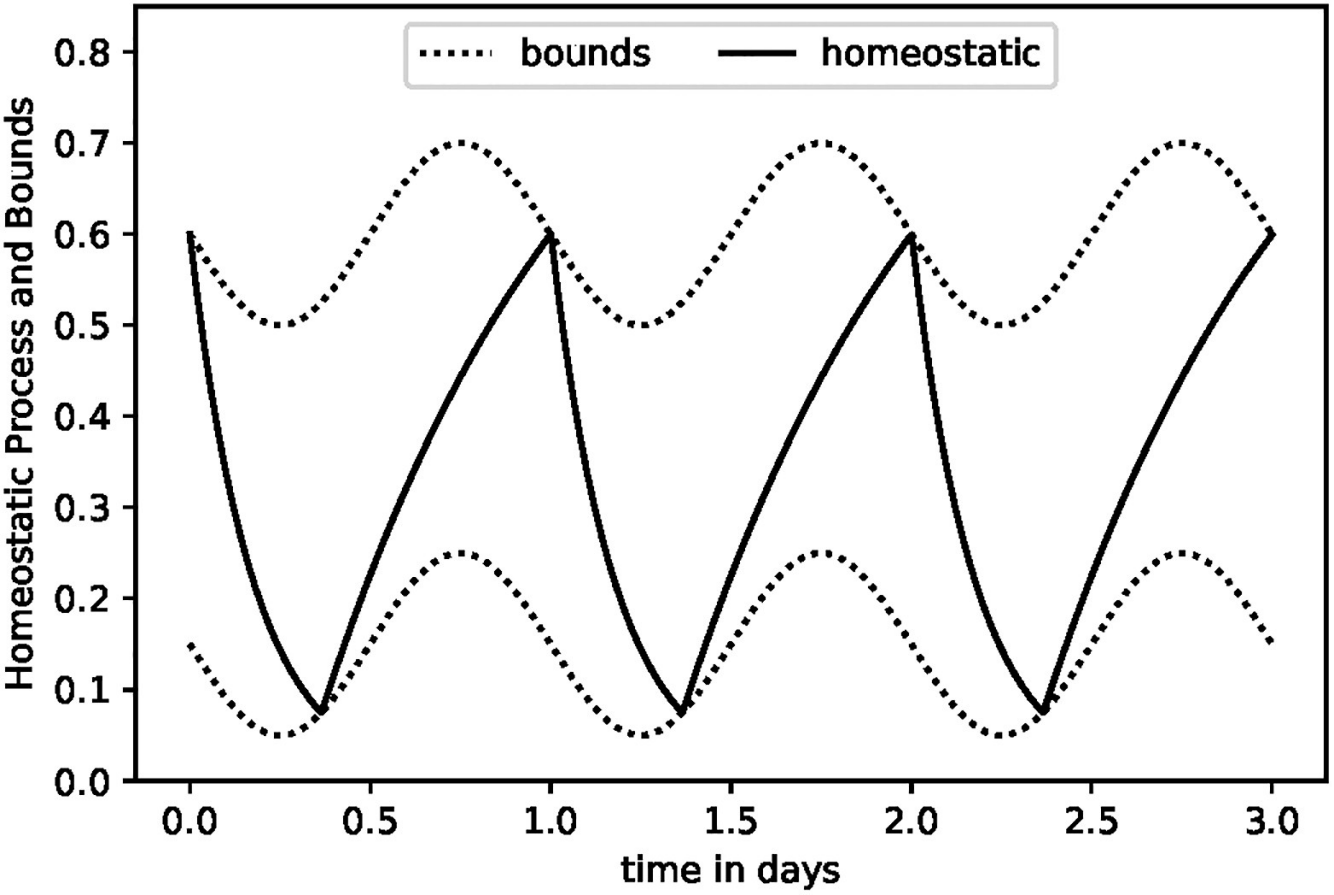
Two-process model.

If the subject is awake, *H*_*t*_ rises until it reaches the upper bound of $H_{t}^{u}$. At this point the subject sleeps and *H*_*t*_ falls until it reaches the lower bound $H_{t}^{l}$. This is illustrated in Fig 1.

### A generalized model

In the two-process model, the twin circadian bounds (*H*^*u*^ and *H*^*l*^) determine the possibility of polyphasic sleep. Varying the parameters may admit the possibility for multiple sleep spells within a circadian cycle. When the homeostatic cycle is very steep (sleep pressure builds rapidly when awake and declines rapidly when asleep), the individual will cycle rapidly between sleeping and waking.

Now assume there is an impediment or cost to switching between sleep and waking. In this case, rather than switching instantaneously, the subject when waking would stay awake until the cost of switching to sleep fell below the loss due to distance from the central cycle. As we show below, this would lead to bounds on the homeostatic process very similar to those in the two-process model. In fact, if the switching cost were constant this would give us the exact upper and lower bounds hypothesized in that model. Both our model and the two-process model ignore the deeper cycles within the circadian cycle, such as REM and non-REM sleep. The physiological necessity of prolonged, restorative sleep justifies the assumption of a fixed cost of waking. A similar justification for non-sleep utility is that productive work (foraging or other work) also cannot be efficiently done in short bursts of activity and requires extended periods of time.

In addition, our model emphasizes the utility derived from waking activities, where utility is a general economic term used to define the well-being or welfare of a subject. Sleep restores physical and mental function to an optimal level, but at the cost of foregoing other activities which are also beneficial. Deviations from the mechanical sleeping behavior suggested by a model of circadian utility are common. Why might a subject be reluctant to sleep even when the homeostatic process is above the circadian cycle? For animals, hunger could be such a motivating force. The subject may choose to remain awake and search for food because the benefit of eating exceeds the cost of lost sleep. We note in the appendix that the two-process model can be interpreted as having an implicit fixed opportunity cost. However, that model cannot be used when the opportunity cost changes over time.

In general this opportunity cost will not be constant, but may vary with the circadian cycle, and with external factors such as time of day or random acts of nature. For example, a natural disaster like a fire or flood would greatly increase the cost of sleeping and alter optimal sleep timing. Seasonal fluctuations in weather and the availability of food would also affect sleep.

Suppose that instead of hard upper and lower bounds on the homeostatic process as conjectured in the two-process model, the subject faced gradually increasing pressure to sleep or wake as distance of the homeostatic process from a single, central circadian cycle increased. Including an opportunity cost of sleep in the form of waking utility allows the subject to balance the gains from productive work (or leisure) against utility losses from sleep. The result is “soft” boundaries that vary with the value of waking activities.

To formalize this intuition, we propose a utility function, *U*, with two components: one, *U*^*y*^, based on adherence to the circadian cycle and another, *U*^*w*^, based on non-circadian benefits. These are shown in the following:

$$\begin{array}{cc} U & =U^{y}\left(H_{t},y_{t}\right)+\chi U^{w}\left(H_{t},A_{t}\right)\\ U^{y}\left(H_{t},y_{t}\right) & =-|H_{t}-y_{t}{|}^{\kappa };\;\kappa >0\\ U^{w}\left(H_{t-1},A_{t}\right) & =\left(1-A_{t}\right){\left[e^{z_{t}}\xi \left(\mu_{W}-H_{t-1}\right)\right]}^{\gamma } \end{array}$$ (3)

The first component is based on a measure of distance of the homeostatic process (*H*_*t*_) from the central circadian cycle (*y*_*t*_), while the second captures benefits from waking activity. The utility parameter *χ* is the relative weight given to *U*^*w*^. Note that as the parameter *κ* in *U*^*y*^ approaches infinity, adherence to the circadian cycle dominates and we get the two-process model from the previous section as a special case. This is because the disutility, *U*^*y*^, goes to zero if |*H*_*t*_ − *y*_*t*_| < 1, and it goes to infinity if |*H*_*t*_ − *y*_*t*_| > 1. The sleep state *A*_*t*_ is a binary variable equaling one when asleep and zero when awake. Non-circadian utility, *U*^*w*^, will depend on waking activities such as eating or providing shelter, which are, in turn, functions of non-sleep time. It is also a function of the homeostatic variable which is a measure of ‘tiredness’. We use the previous period’s value *H*_*t*−1_ since this is the level that will matter for exploiting opportunity costs. As *H*_*t*−1_ rises toward its upper bound, *μ*_*W*_, the subject becomes less able to perform well at waking activities. The parameters *ξ* and *γ* modify the benefits from waking activities. Non-circadian utility while sleeping is assumed to be zero. However, there are dynamic benefits, since sleeping reduces tiredness and this is useful in the future when the subject wakes. Opportunity cost is given by the environmental state variable, *z*_*t*_, which we assume follows an autogressive process.

Without loss of generality we can reformulate the homeostatic process as shown in Eq (2). We reconfigure the equation so that the upper asymptote is *μ*_*W*_ and the lower one is *μ*_*S*_, with *μ*_*W*_ > 0 > *μ*_*S*_:

$$\begin{array}{c} H_{t+1}=\{\begin{array}{cc} \mu_{W}+\left(H_{t}-\mu_{W}\right)e^{-1/\nu_{W}} & \text{if}\;A_{t}=0\\ \mu_{S}+\left(H_{t}-\mu_{S}\right)e^{-1/\nu_{S}} & \text{if}\;A_{t}=1 \end{array} \end{array}$$ (4)

**Fig 2:**
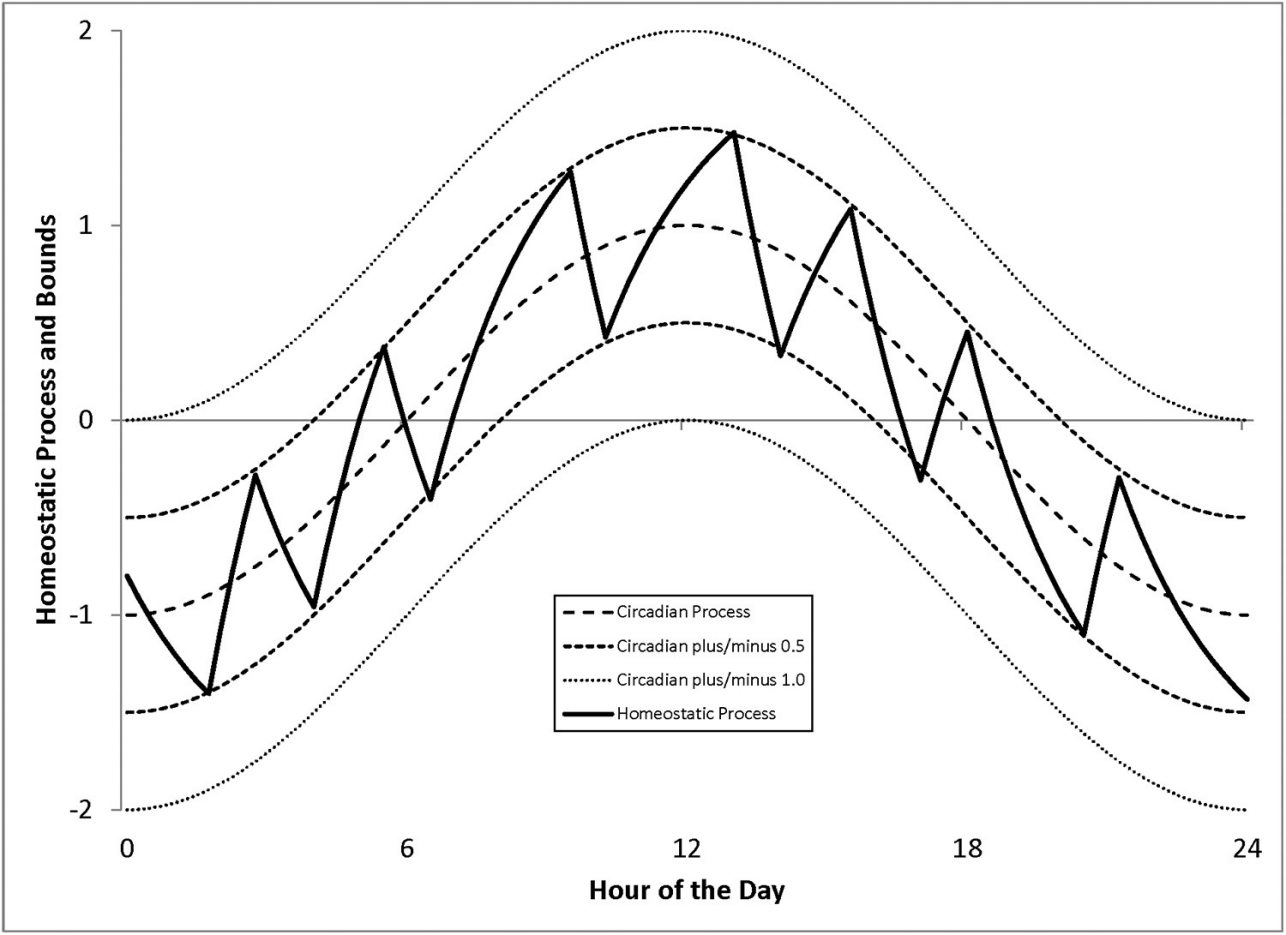
Behavior of the generalized model. Fixed Switching Cost Only.

**Fig 3:**
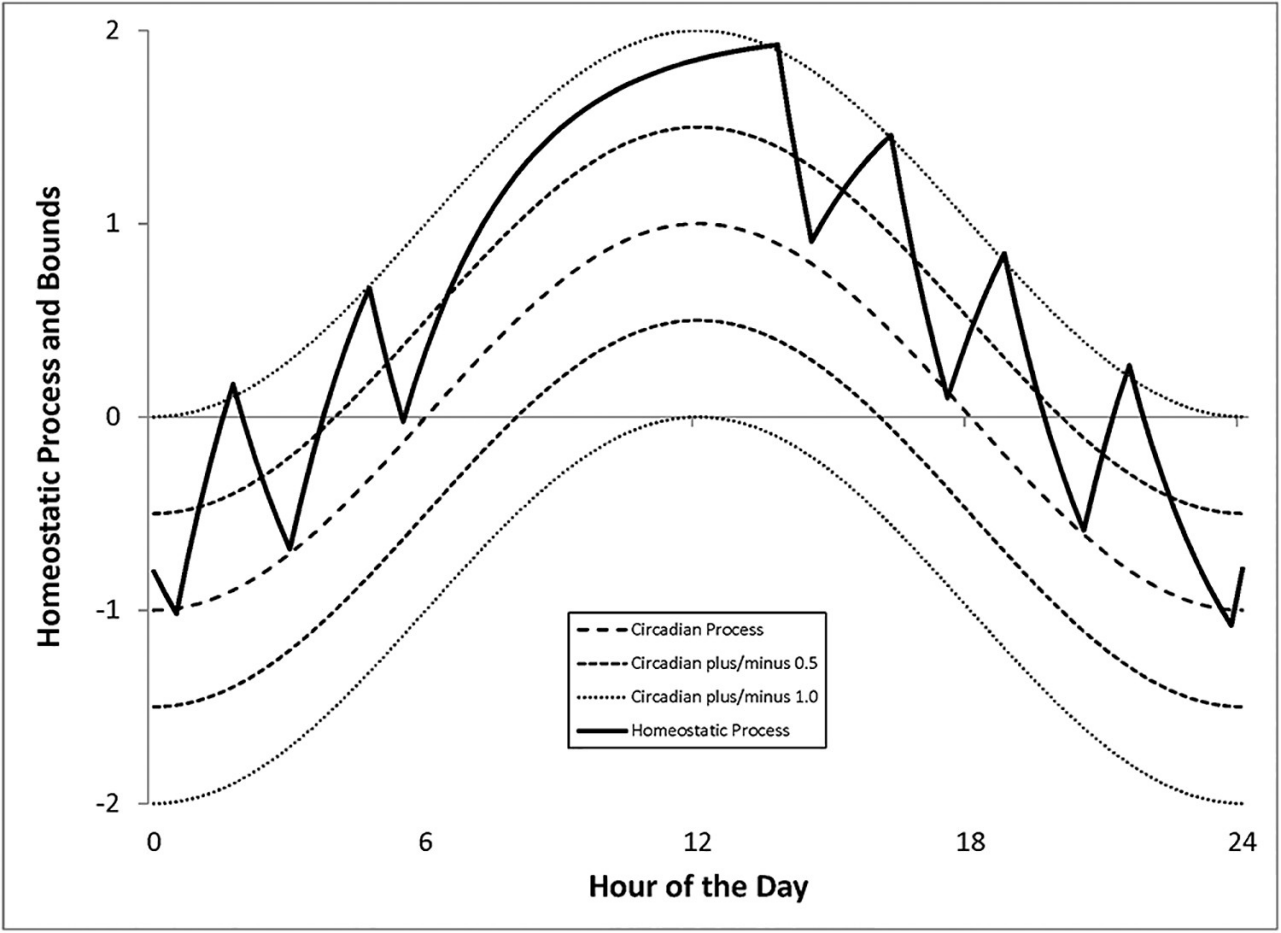
Behavior of the generalized model. Fixed opportunity cost of sleep.

**Fig 4:**
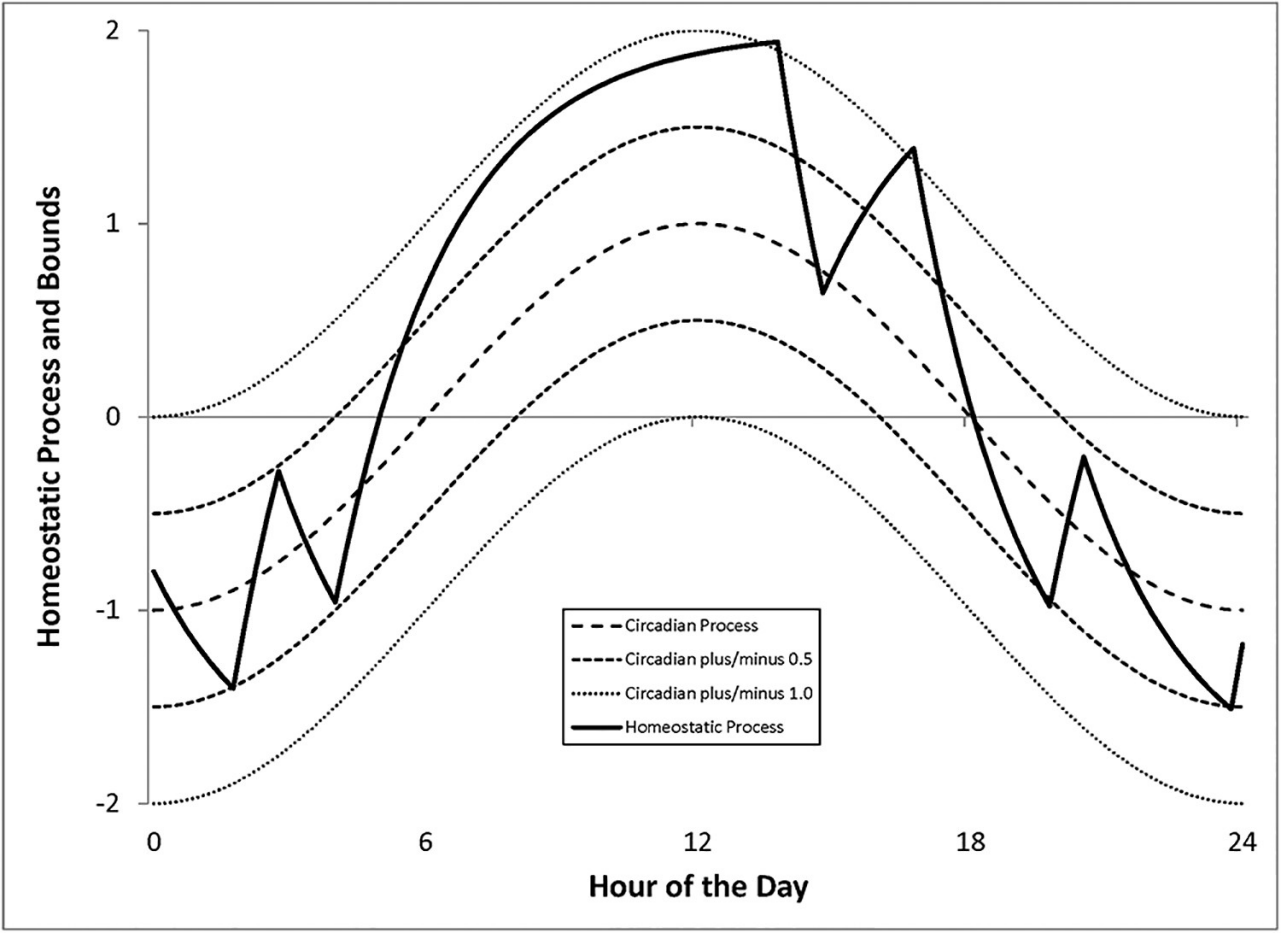
Behavior of the generalized model. Time-varying opportunity cost of sleep.

Figs 2–4 illustrate the behavior of our model. In each figure the central circadian cycle is shown by the long dashed line. A small utility threshold away from this central cycle is shown by the two medium dashed lines. A large utility threshold is shown with the short dashed lines. The behavior of the homeostatic process is illustrated with the solid line.

Fig 2 shows the behavior with a fixed cost of switching between sleep and waking only. There is no opportunity cost so the subject cares only about circadian utility, switching between waking and sleeping only when the circadian disutility is larger than the small switching cost.

Fig 3 illustrates the behavior with an opportunity cost of sleep. The subject will switch from waking to sleep when the circadian disutility is greater than the switching cost plus the opportunity cost. They will switch from sleep to waking when the circadian disutility is greater than the switching cost *minus* the opportunity cost.

Fig 4 shows behavior when the opportunity cost varies over the course of the day. In this example there is zero opportunity cost between 6 p.m. and 6 a.m., but the opportunity cost is positive from 6 a.m. to 6 p.m. To model nocturnal animals, we could simply change the timing of productive waking hours from day to night.

## Modeling sleep in animals

There is an extensive literature on sleep patterns of animals. One early summary of this literature is [8] which reports sleep duration for 150 species drawn from 200 studies. A more recent study by [9] surveys the sleep behavior of insects, fish, amphibians, reptiles, birds and mammals. [10] show how a single model with different parameter values can explain variations in sleep patterns across seventeen mammal species. Almost all of these studies involved captive animals, however, and subsequent research has shown that captive sleep behavior can differ markedly from sleep behavior in the wild. Nearly 50 years ago, [11] pointed out that the duration of sleep among animals should not be considered in isolation, but instead should be considered together with other important factors of the animals’ biological make up. We would therefore expect most animals in the wild to sleep less than their counterparts in captivity. Sleep studies involving wild animals are difficult to conduct and are still relatively rare, though advances in monitoring technology make them increasingly feasible.

In spite of technological barriers, for some animals data is available that permit comparisons for a species in the wild and in captivity. [12] report the study of two wild African elephant matriarchs and find an average daily sleep of 2.20 hours as compared with the 6.28 hours reported for captive zoo elephants from [13]. [14] and [15] examined the sleep behavior of three-toed sloths in the wild. They report that sloths in the wild sleep 9.63 hours per day on average. This is much less than the 15.85 hours reported by [16] for captive sloths. Elephants and sloths are interesting examples to consider because elephants are short sleepers and sloths are long sleepers.

We assume that herbivorous animals, such as elephants, use their time to either sleep or to forage. If the animal sleeps, the value of *H*_*t*_ will fall, but no non-circadian utility is generated. If the animal is awake it will forage and foraging generates consumption according to a mathematical representation we will refer to as a production function. This, in turn, generates utility. In our model foraging includes all activities which generate well-being for the animal, not just the search for food. This includes avoiding predation, caring for young, and even play.

Our model is agnostic on whether animals consciously choose sleep or not. Our formulation neither requires nor excludes that possibility. We treat the circadian utility described above as a given; animals are endowed with this as part of their genetic heritage. Forward-looking behavior can occur even if the animal has no concept of the future and does no explicit planning. All that is required is that it be aware of its environment and be able to infer the opportunity cost of sleep, which will consist primarily of the likely reward to searching for food and avoiding predation.

The circadian cycle is given by the following simple function:

$$y_{t}=-\cos(\frac{t2\pi }{q})$$ (5)

where *q* is the number of periods in a day.

Foraging productivity depends on the stock of sleep coming into the period, *H*_*t*−1_ (which was determined last period) and the environmental state, *z*_*t*_. Circadian utility, however, depends on this stock now, which is its past value plus the increment upward or downward due to waking or sleeping today. Finally, there is a fixed utility cost of λ for transitioning from waking to sleeping and back again. This is illustrated in the equation below, where we have substituted Eq (4) into the circadian utility function. Hence, the subject comes into the period knowing *H*_*t*−1_ and *A*_*t*−1_, chooses *A*_*t*_, and *H*_*t*_ evolves given this choice. We model the animal as solving the following:

$$\begin{array}{c} \begin{array}{cc} \max_{A_{t}}U & =A_{t}{\left[e^{z_{t}}\xi {\left(\mu_{W}-H_{t-1}\right)}^{\eta }\right]}^{\gamma }\\ & -\chi [\begin{array}{c} \left(1-A_{t}\right){\left|\mu_{W}+\left(H_{t-1}-\mu_{W}\right)e^{-1/\nu_{W}}-y_{t}\right|}^{\kappa }\\ +A_{t}{\left|\mu_{S}+\left(H_{t-1}-\mu_{S}\right)e^{-1/\nu_{S}}-y_{t}\right|}^{\kappa } \end{array}]\\ & -\lambda |A_{t}-A_{t-1}| \end{array} \end{array}$$

We assume that the portion of foraging production related to the environment follows a stochastic autoregressive process,

$$\begin{array}{c} z_{t}=\rho^{\frac{1}{q}}z_{t-1}+\sigma \varepsilon_{t}, \end{array}$$ (6)

where *ρ* is the measure of autoregressive reversion to the mean measured in per hour terms and *ε*_*t*_ are independent and identically distributed random variables with mean zero and variance one.

The choice of *A*_*t*_ is a binary one; there is no marginal condition. Instead, the agent chooses the value of *A*_*t*_ which generates the greatest utility given current values for *H*_*t*_, *y*_*t*_ and *z*_*t*_.

### Elephant behavior

We now illustrate this model with an application to elephants. [12] monitored the sleep behavior of two African Elephant matriarchs for 35 days using an activity data logger implanted subcutaneously in their trunks. Matriarch 1 averaged 2.3 hours of sleep per day, while Matriarch 2 slept an average of 1.8 hours. The standard deviations of sleep time were 1.3 and 1.0 hours. Both were polyphasic sleepers; Matriarch 1 sleeping an average of four spells per day and Matriarch 2 sleeping for five spells. Both had one main sleep episode averaging 1.3 and 0.8 hours, respectively. A number of shorter sleep episodes occurred usually before the main episode.

[13] observed the sleep patterns of two groups of captive female Asian Elephants; one group at a zoo and the other at a circus. A total of 294 elephant-nights of sleep were observed over both groups. Sleep was determined by examining videotape. Zoo elephants slept an average of 6.28 hours per night with a standard deviation of 0.21. Circus elephants slept 3.52 hours per night with a standard deviation of 0.17. They also found polyphasic sleep noting, “In all animals the hourly amount of sleep gradually increased during the course of the night, reaching a maximum between approximately 0100 and 0400 hours. The largest amounts of sleep invariably occurred after midnight.”

We note that wild elephants slept only a third as many hours as captive elephants, and that the variability of time slept was five times as large. We hypothesize that wild elephants not only have higher opportunity costs of sleep time, but that the cost is also much more variable over time. Since the primary opportunity costs are likely time spent foraging for food and avoiding danger, it makes sense that these costs are both higher and more variable for elephants in the wild.

We attempt to match the sleep patterns of wild and captive zoo elephants using our model. The model has a large number of parameters. Some of these are free parameters and can be chosen only by fitting model behavior to observed data. However, many of these are not free and can be assigned values outside of a data-fitting exercise. Table 1 lists the model parameters.

**Table 1:** Model parameters, predicted and actual sleep for elephants.

Parameter	Description	Captive	Wild
*q*	periods per day	144	
*κ*	curvature of circadian penalty	2.0	
*η*	curvature of foraging production	1.0	
*γ*	curvature of foraging utility	0.8	
*ρ*	autoregressive component of *z*_*t*_	0.9	
*ν*_*W*_	decay half-life while awake	8 hours	
*ν*_*S*_	decay half-life while asleep	8 hours	
*μ*_*W*_	waking asymptote	1.0	
*μ*_*S*_	sleeping asymptote	-0.9056	
λ	wake-sleep switching cost	0.2	
*χ*	utility weight on circadian cycle	10.0	
*ξ*	opportunity cost of sleep	1.0	6.619
*σ*	standard deviation of innovations to *z*_*t*_	0.00576	0.12172
$\bar{S}$	model mean hours of sleep	6.278	2.197
*σ*_*S*_	model standard deviation	0.205	1.031
	observed mean hours of sleep	6.280	2.200
	observed standard deviation	0.210	1.030

We calibrate our model as follows: *q* is the number of model periods per day and is set to 144, giving periods of 10 minutes in length. *κ* is the curvature of the penalty for the homeostatic process deviating from the circadian cycle. We set this equal to 2.0, which imposes a simple quadratic penalty. *η* is the curvature of foraging production in response to sleep. We assume that there is no diminishing marginal product of sleep and set this value to 1.0. *γ* is the curvature of the non-sleep utility function. In line with values typically used in the economics literature, we set this to 0.8. *ρ* is the autoregressive coefficient for the environmental state, *z*_*t*_. We set this to 0.9 per hour which generates a large amount of persistence over time. All these values are held fixed across both species and wild versus captive animals.

Some other parameter values are constant across our simulations, but are harder to pin down. In these cases we conduct sensitivity analysis to see how our results depend on these choices. *ν*_*W*_ and *ν*_*S*_ are the decay half-lives associated with the homeostatic process while awake and asleep. We set these both to 8.0 hours. *μ*_*W*_ and *μ*_*S*_ are the asymptotic bounds for the homeostatic process when waking and asleep. Parameter values in utility and production functions allow us to normalize one of these bounds. The other is a free parameter. For elephants, which sleep little, we normalize *μ*_*W*_ to 1.0 and leave *μ*_*S*_ free. Sloths sleep much more and in that case we normalize *μ*_*S*_ to 1.0. λ is the cost of switching from awake to sleeping or sleeping to awake. If this value is too high, subjects would remain always awake or asleep. If it is too low, they will engage in bouts of micro sleep; awake one moment, asleep the next, then awake again. We set this value to 0.2, which generates a realistic pattern of polyphasic sleep for both species. *χ* is the relative weight of sleep utility to that of foraging utility. We set this to 10.0.

Finally, we have free parameters to calibrate. For captive animals we normalize *ξ*, the opportunity cost of sleep, to 1.0. We choose values for *μ*_*S*_ and *σ* (the standard deviation of stochastic environmental shocks, *z*_*t*_) to match the daily mean and standard deviation from the data for capitve elephants to that for a simulation of our model over 100,000 days. We then set this value of *μ*_*S*_ and proceed to match the behavior of wild elephants by changing the values of *ξ* and *σ* to match their daily mean and standard deviation. These parameter values and results of the simulations are also reported in Table 1.

In our benchmark simulation, we find that relative to captive zoo elephants, wild ones face an opportunity cost of time *ξ* that is on average 6.6 times greater. In addition, the volatility of the randomness in opportunity cost is thirteen times higher.

**Fig 5:**
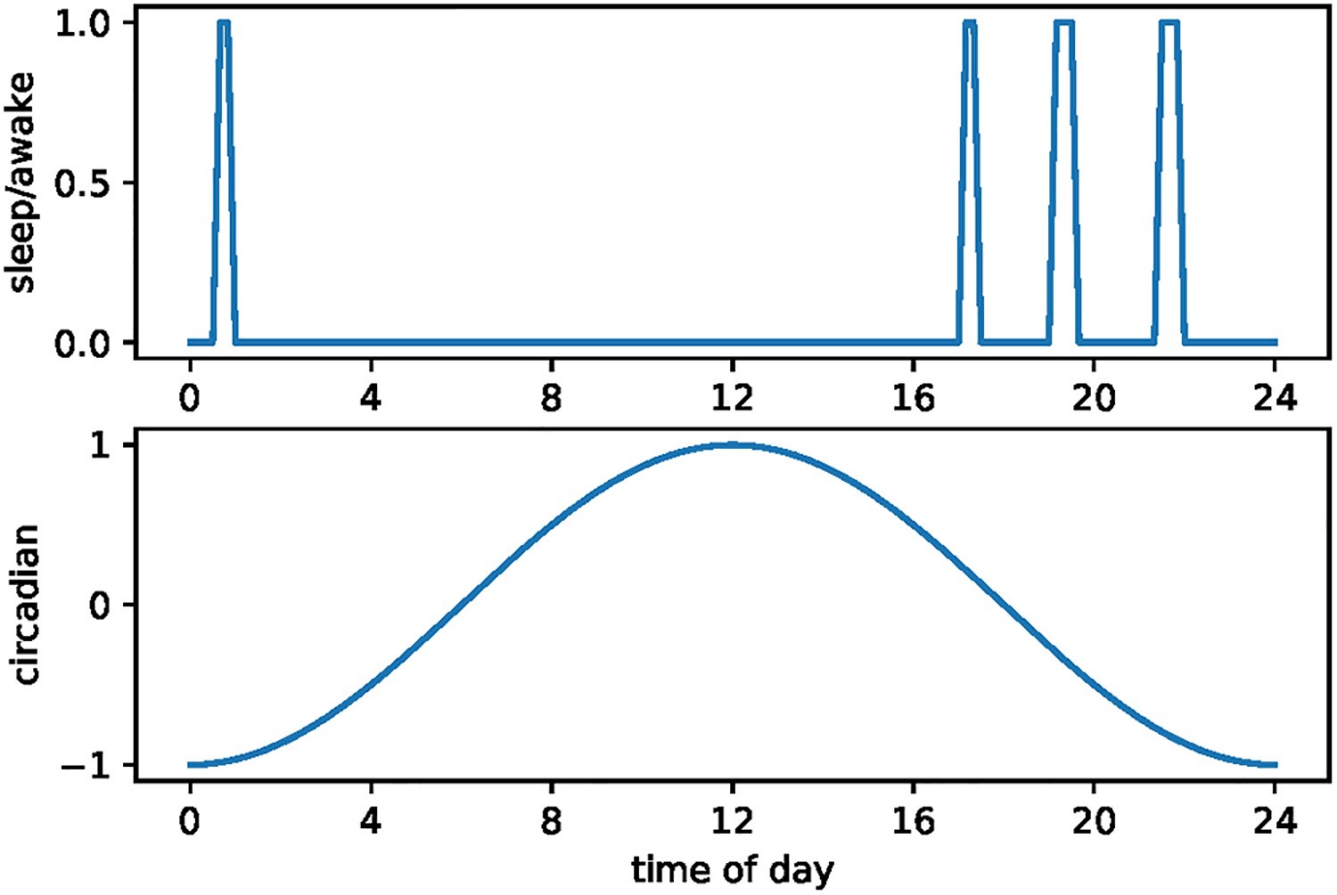
Stylized elephant sleep patterns.

Our model also generates polyphasic sleep roughly in line with that for wild elephants. Fig 5 shows the sleep patterns for a stylized case where there are no random shocks to foraging productivity. In this case the elephant sleeps in four equally-spaced episodes over the night. In the wild the episodes are not equally spaced, but we are able to match the number of episodes by choosing a value of λ (sleep/wake switching cost) of 0.2. For comparison, the difference in the maximum and minimum for total utility per period over the course of a day is 29.35.

What do we learn from this exercise about elephant sleep? Our model fits the observed sleep behaviors of wild and captive elephants remarkably well. We also find elephants in the wild have an opportunity cost of sleep that is 6.6 times greater than that of captive zoo elephants. The opportunity cost difference sheds light on why elephants in the wild sleep less than elephants in captivity.

### Three-toed sloth behavior

We next apply our model to three-toed sloths. [14] conducted a study of Three-Toed Sloths in the wild. Their sample was three wild females who were captured and fitted with electroencephalogram (EEG) and electromyogram (EMG) recorders. A total of 12.8 days of essentially continuous recording were obtained. A second study examined two sloths over a 7-month period using radio collars and the automated radio-telemetry system (ARTS). They found that sloths in the wild sleep 9.63 hours per day on average with a standard deviation of 0.5 hours. Sloths are highly polyphasic sleepers with a tendency and to be awake during the first two-thirds of the night and asleep during the last. A typical sleep cycle of REM and non-REM sleep lasted 46 minutes.

In contrast, [16] examined a group of 24 captive sloths using similar EEG equipment. Sleep patterns were observed over a period of two months. They found an average sleep time of 15.85 hours per day. They also document a great deal of polyphasic sleep.

As with elephants, we hypothesize that differences in opportunity costs between wild and captive sloths drives the observed differences in sleep patterns. Again we calibrate our model first using data on captive sloths and then determine the level of opportunity cost needed to generate observed sleep patterns in the wild. We use the same calibrated parameters as with elephants except for the values shown in Table 2. The table shows that the opportunity cost for wild sloths is about five time higher than that for captive sloths. We have no data on the standard deviation of sleep time for captive sloths, so we hold the value of *σ* the same over both groups. In a follow-up study to [14], [15] show that differences in wild versus captive sleep patterns cannot be due to predation risk. They examine two groups of sloths, one on the mainland with natural predators and the other on an island with no predators present. They find no statistical difference in hours slept between these two groups. Hence, we conclude that all sleep differences are driven by foraging opportunity costs.

**Table 2:** Model parameters, predicted and actual sleep for sloths.

Parameter	Description	Captive	Wild
*μ*_*W*_	waking asymptote	5.614	
*μ*_*S*_	sleeping asymptote	-1.0	
*ξ*	opportunity cost of sleep	1.0	6.283
*σ*	standard deviation of innovations to *z*_*t*_	0.0384	0.0175
$\bar{S}$	steady state hours of sleep	15.827	9.630
*σ*_*S*_	standard deviation of hours of sleep	0.500	0.498
	observed mean hours of sleep	15.830	9.63
	observed standard deviation	n/a	0.50

**Fig 6:**
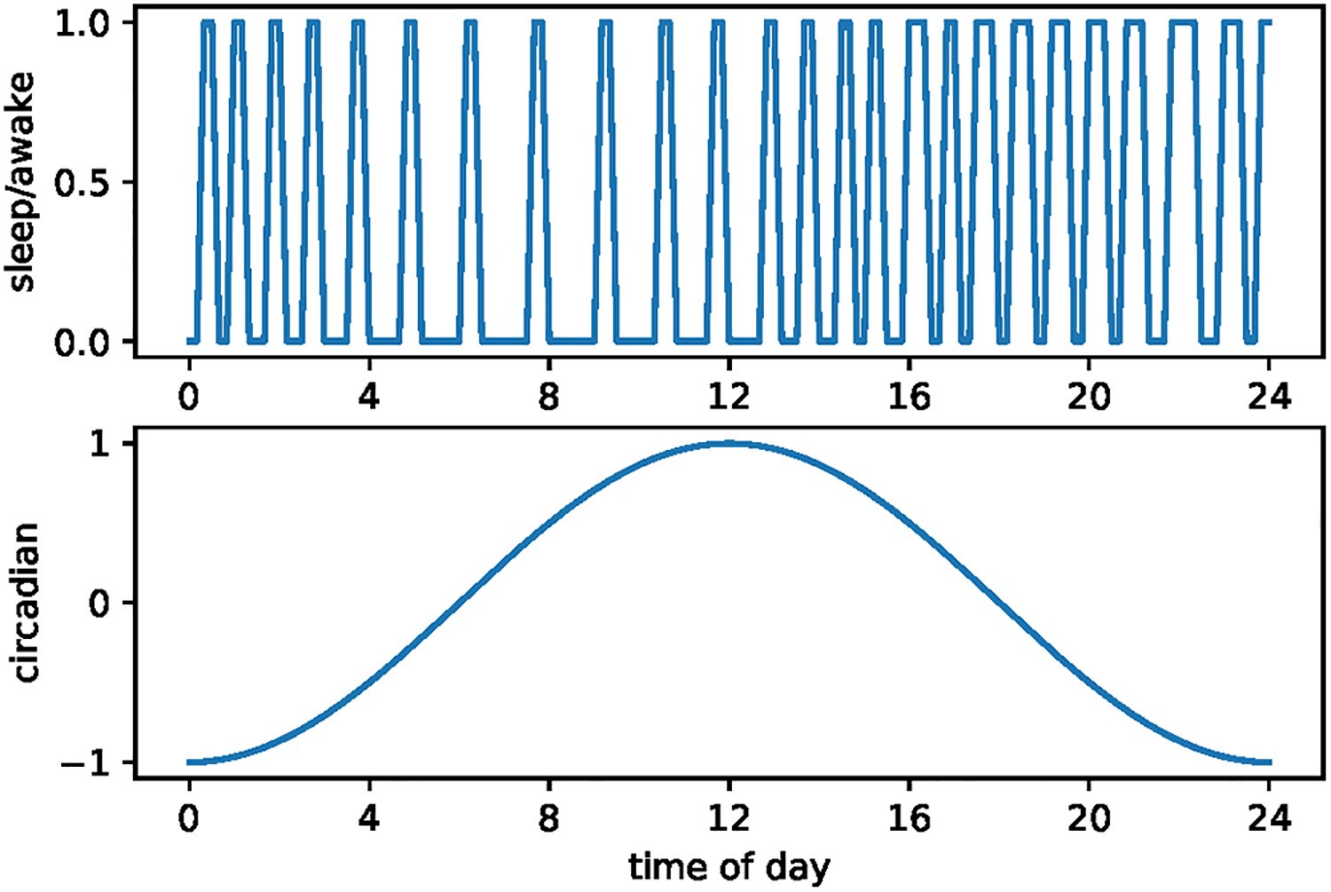
Stylized sloth sleep patterns.

We are also able to generate sleep patterns over the day that match those of wild sloths. Fig 6 shows the timing of sleep for a stylized day with no random shocks to opportunity cost. There are 25 sleep episodes with shorter duration as the circadian cycle rises and longer duration as it falls. As with elephants, the switching cost in our calibration is λ = 0.2, but now the difference between high and low utility over the full 24-hour day is 69.32.

In sum, we find that our model fits the difference between wild and captive sloths quite well. We find that the opportunity cost of sleep for wild sloths is six times greater than for captive sloths, a number remarkably similar to that for elephants.

### Sensitivity analysis

We check the robustness of our results by comparing the relative opportunity costs for different parameterizations of our model. We focus our sensitivity analysis on parameters for which values are not easily found in the existing literature. The parameters are: 1) the half-lives, *ν*_*W*_ and *ν*_*S*_, 2) the weight of sleep in total utility, *χ*, and 3) the switching cost, λ. We look at a change in the degree of diminishing marginal utility in non-sleep utility as measured by *γ*.

Results of the sensitivity analyis are shown in Table 3. For our first alternative parameterization (labeled B) we drop the half-lives for the homeostatic process from 8.0 hours to 4.0 hours. This means that this process converges more quickly to the bounds. As a result the homeostatic process veers away from the circadian cycle more and the subject pays a higher cost for deviating from the circadian cycle. This means a higher opportunity cost of sleep is required to rationalize observed sleep patterns. For elephants, the opportunity cost for wild animals more than doubles from 6.619 to 14.103. For sloths it increases by 40 percent from 6.283 to 8.847.

**Table 3:** Sensitivity analysis.

Scenario	Parameter change	Relative opportunity cost of sleep, *ξ*	
		Elephants	Sloths
A	benchmark	6.619	6.283
B	lower half-lives	14.103	8.847
C	higher sleep weight	16.744	9.400
D	lower switching cost	6.876	6.332
E	no diminishing utility	6.410	4.585

For our second alternative (labeled C) we increase the weight in total utility from 10.0 to 20.0. This should yield results that are qualitatively the same as lowering half-lives. Table 3 confirms this with elephant opportunity cost rising to 16.744 and sloth opportunity cost rising to 9.400.

**Fig 7:**
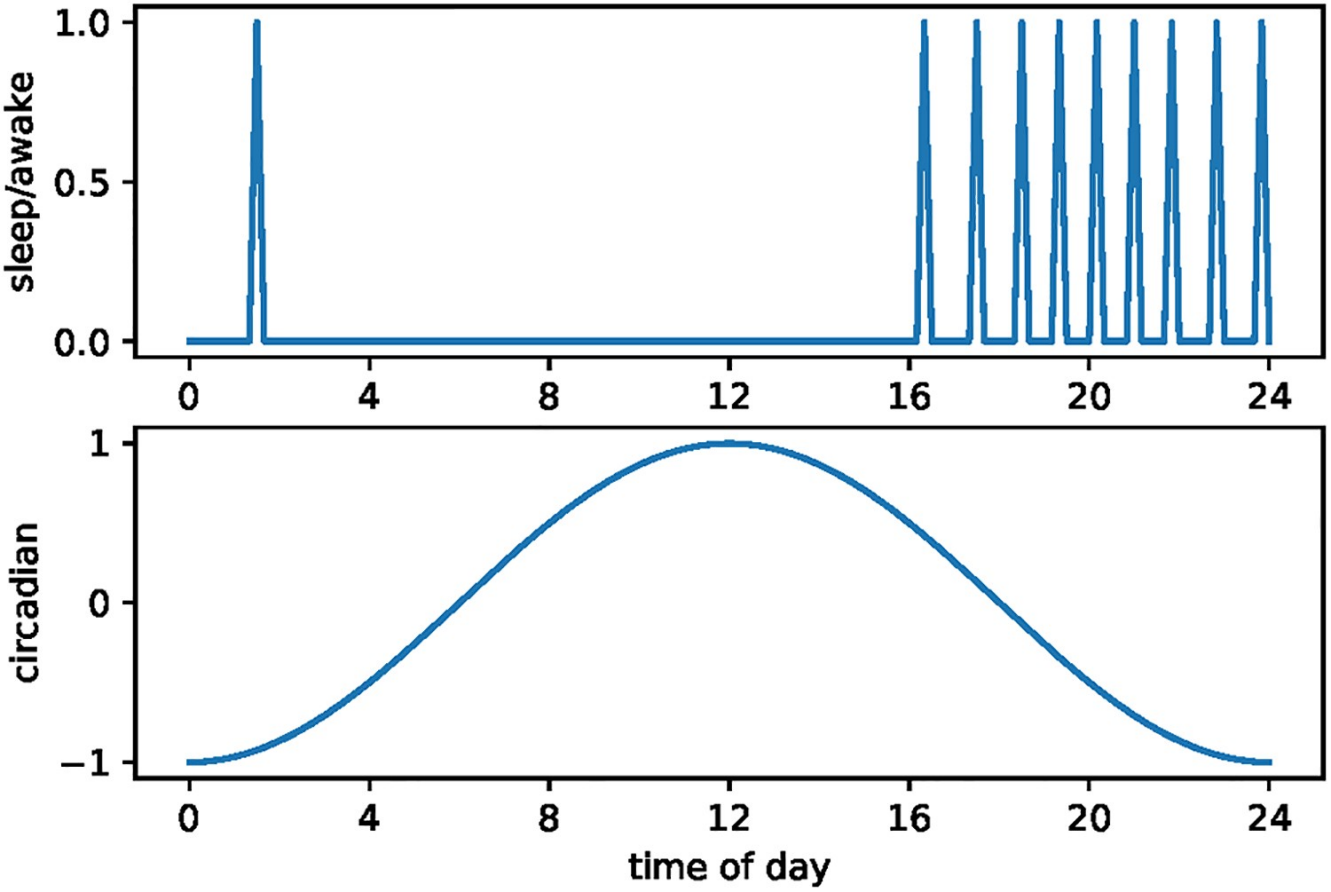
Stylized elephant sleep patterns with low switching cost.

We lower the cost of sleep switching from 0.2 to 0.02 for our third alternative, D. In this case we observe little change in the implied opportunity cost for wild animals. However, as expected, we do observe a large increase in polyphasic sleep. Contrast Fig 7 with the benchmark case in Fig 5. The number of sleep episodes increases from four to ten.

Lastly, we change the value of *γ* from 0.8 to 1.0 which we label E. This makes non-sleep utility linear in the amount of foraging done. In economic terms it means that marginal utility–the change in utility as foraging production changes–does not diminish as more foraging output goes up. There is little change for the implied opportunity cost for elephants, but the cost falls by 27 percent to 4.585.

As expected, altering the relative cost of sleep as in scenarios B and C, raises the imputed opportunity costs. However our costs are more robust for the other two scenarios. Even when the opportunity costs change substantially, they do not do so by orders of magnitude. The largest change is scenario C for elephants where the cost rises by a factor of 2.53. We view this as cause for cautious optimism regarding the usefulness of the model. Despite the small amount of data relative to the number of model parameters, the model generates opportunity cost estimates that do not vary wildly across the choices of parameter values.

## Conclusions

This paper introduces a new model of sleep for animals. It extends the classic two-process model of sleep to account for differences in external circumstances. We apply this model to previously-collected data on elephants and sloths, comparing sleep patterns in the wild with sleep patterns in captivity. We find that the model does well in explaining sleeping patterns for both types of animals, in both the captive state and in the wild state.

The model is sufficiently flexible that it can be applied to all animal life forms, including humans. The key insight is that sleep is not a purely biological function, but is also a function of the immediate environment and circumstances of the animal. The direct inference from this is that sleep is a dynamic process; it will vary depending on whether an animal is in captivity or in the wild, but it will also likely vary across the year, depending on the external circumstances the animal faces. For example, our model predicts that predators with fixed territories will have very different sleep patterns when migrating prey are abundant compared to when they are scarce. Any measurement of sleep will be conditional on the particular conditions.

Understanding the role opportunity cost plays is important to understanding sleep patterns in animals and in people. This is particularly true in assessing sleep health. Because it ignores benefits other than those accruing from sleep, a purely mechanical model of sleep, like the two-process model, will prescribe optimal sleep patterns that are not consistent with changes in the environment and therefore are not truly optimal. The cost of lost sleep may be more than offset by the benefits of increased consumption of food or avoidance of predators. Sleep may be driven primarily by instinct, but that instinct is the result of evolutionary pressure in the face of environments where opportunity cost can and does change.

## Appendices

### Fixed opportunity cost is implicit in the two-process model

With a small switching cost, the subject will sleep and *H*_*t*_ will fall until it reaches the lower bound. Then the subject will wake and *H*_*t*_ will rise until it reaches the upper bound. Suppose *μ*_*W*_ = −*μ*_*S*_ and *ν*_*W*_ = *ν*_*S*_. Then the subject will sleep exactly half the time. The reason is that the homeostatic process moving upward (while awake) is perfectly symmetric with the process moving downward (while sleep).

Now suppose we add an opportunity cost of sleeping. We can think of circadian utility as some measure of the distance between *H*_*t*_ and *y*_*t*_, i.e. *u* = *u*(*H*_*t*_ − *y*_*t*_). We are now adding an additional amount of utility that derives simply from being awake. Suppose this is a constant, $\bar{u}$. If sleeping, the subject will remain sleeping until the disutility from *H*_*t*_ falling below *y*_*t*_ *plus* the opportunity cost is greater than the cost of waking up. In the two-process model this would be achieved by increasing the value of *μ*_*S*_, so that the lower bound is reached sooner. Similarly, if waking, the subject remains awake until the circadian cost of waking plus $\bar{u}$ is less than the switching cost, and the upper bound is reached later. This is achieved by raising the value of *μ*_*W*_.

Raising *μ*_*W*_ and *μ*_*S*_ will result in less sleep and more time awake. Hence, the value of the homeostatic process asymptotes relative to the peaks and troughs of the circadian cycle (normalized to 1 and -1) is what reflects the implicit opportunity cost in the two-process model.

### Calibration details

We match the mean and standard deviation of daily hours slept in our model to data collected on wild and captive animals. We do so by choosing two parameter values. For captive animals these are either *μ*_*S*_ or *μ*_*W*_ along with *σ*. For wild animals we choose *ξ* and *σ*.

We use the following process. First, we draw a series of 14,400,000 random draws from a standard normal distribution. We save these draws and use them in each simulation. We then proceed to simulate our model for some starting guesses of the two parameter values. For each 10-minute period in the simulation the subject is either awake or asleep. When the simulation is finished, we divide the data up into 100,000 days and take the average and standard deviation of hours slept per day. We compare these with the relevant values from animal studies. We use the SciPy package in Python to minimize the sum of absolute differences between the simulation and the data. The fmin function in SciPy uses a Nelder-Mead simplex algorithm to find the minimum for this sum of two absolute differences.

## References

[1] Walker MP (2017) Why we sleep: Unlocking the power of sleep and dreams. New York: Scribner, an imprint of Simon & Schuster, Inc.

[2] Carskadon MA, Dement WC (2011) Monitoring and staging human sleep In: Kryger MH, Roth T, Dement WC, editors. Principles and practice of sleep medicine. 5th ed Elsevier pp. 16–26.

[3] Borbély AA (1982) A two process model of sleep regulation. Human Neurobiology 1(3):195–204. 7185792

[4] Borbély AA, Achermann P (1999) Sleep homeostasis and models of sleep regulation. Journal of Biological Rhythms 14(6):559–570. 10.1177/074873099129000894 10.1177/074873099129000894 10643753

[5] Rempe MJ, Best J, Terman D (2010) A mathematical model of the sleep/wake cycle. Journal of mathematical biology 60(5):615–644. 10.1007/s00285-009-0276-5 19557415 10.1007/s00285-009-0276-5

[6] Cardon JH, Eide ER, Phillips KL, Showalter MH (2018) A model of sleep, leisure and work over the business cycle. Journal of Economic Dynamics and Control 95:19–36. 10.1016/j.jedc.2018.08.003

[7] Skeldon AC, Dijk DJ, Derks G (2014) Mathematical models for sleep-wake dynamics: Comparison of the two-process model and a mutual inhibition neuronal model. PLOS ONE 9(0):e103877 10.1371/journal.pone.0103877 25084361 10.1371/journal.pone.0103877 PMC4118955

[8] Campbell SS, Tobler I (1984) Animal Sleep: A review of sleep duration across phylogeny. neuroscience and behavioral reviews 8:269–300. 10.1016/0149-7634(84)90054-X 10.1016/0149-7634(84)90054-x 6504414

[9] Siegel JM (2008) “Do all animals sleep?”. Trends in Neurosciences 31(4):208–213. 10.1016/j.tins.2008.02.001 18328577 10.1016/j.tins.2008.02.001 PMC8765194

[10] Phillips AJ, Robinson PA, Kedziora DJ, Abeysuriya RG (2010) Mammalian sleep dynamics: how diverse features arise from a common physiological framework. PLOS Computational Biology 6(6):e1000826 10.1371/journal.pcbi.1000826 20585613 10.1371/journal.pcbi.1000826 PMC2891699

[11] Hediger H (1969) Comparative Observations on Sleep. Proceedings of the Royal Society of Medicine 62:153–156. 5775231 10.1177/003591576906200213 PMC1810747

[12] Gravett N, Bhagwandin A, Sutcliffe R, Landen K, Chase MJ, Lyamin OI, et al (2017) Inactivity/sleep in two wild free-roaming African elephant matriarchs—Does large bodysize make elephants the shortest mammalian sleepers? PLOS ONE 12(3): e0171903 10.1371/journal.pone.0171903 28249035 10.1371/journal.pone.0171903 PMC5382951

[13] Tobler I (1995) Is sleep fundamentally different between mammalian species? Behavioural Brain Research 69:35–41. 10.1016/0166-4328(95)00025-O 7546316 10.1016/0166-4328(95)00025-o

[14] Rattenborg N, Voirin B, Vyssotski A, Kays R, Spoelstra K, Kuemmeth F (2008) Sleeping outside the box: Electroencephalographic measures of sleep in sloths inhabiting a rainforest. Biology Letters 4:402–405. 10.1098/rsbl.2008.0203 18482903 10.1098/rsbl.2008.0203 PMC2610152

[15] Voirin B, Scriba MF, Martinez-Gonzalez D, Vyssotski AL, Wikelski M, Rattenborg NC (2014) Ecology and neurophysiology of sleep in two wild sloth species. Sleep 37(4):753–761. 10.5665/sleep.3584 24899764 10.5665/sleep.3584 PMC4044746

[16] Galvao de Moura Filho AG, Huggins SE, Lines SG (1983) Sleep and waking in the three-toed sloth, Bradypus Tridactylus Comparative Biochemistry and Physiology Part A: Physiology 76(2):345–355. 10.1016/0300-9629(83)90336-5 6139209

